# The efficacy and safety of Xiao-Ban-Xia-Tang in the treatment of chemotherapy-induced nausea and vomiting: A systematic review and meta-analysis

**DOI:** 10.3389/fphar.2024.1393597

**Published:** 2024-06-12

**Authors:** Ling Li, Shangmei Jia, Chenghao Yu, Shasha Shi, Fu Peng

**Affiliations:** ^1^ State Key Laboratory of Southwestern Chinese Medicine Resources, Department of Basic Medicine, Chengdu University of Traditional Chinese Medicine, Chengdu, Sichuan, China; ^2^ Department of Basic Medicine, Shaanxi University of Chinese Medicine, Xianyang, Shaanxi, China; ^3^ Department of West China School of Pharmacy, Sichuan University, Chengdu, Sichuan, China

**Keywords:** chemotherapy, nausea, vomiting, systematic review, Xiao-Ban-Xia-Tang

## Abstract

**Background:**

Chemotherapy-induced nausea and vomiting (CINV) is one of the most frequent and critical side effects due to chemotherapeutics. In China, Xiao-Ban-Xia-Tang (XBXT) has already been applied extensively to prevent and treat CINV. However, there is limited testimony on the effectiveness and safety of this purpose, and there was no correlative systematic review. The aim of this review was to systematically evaluate the effectiveness and safety of XBXT in preventing and treating CINV.

**Methods:**

The systematic search was conducted in eight databases to acquire randomized controlled trials (RCTs) that appraised the effect of XBXT in treating CINV. The vomiting and nausea relief efficiency, eating efficiency, quality of life, and adverse reactions were explored for efficacy assessment. Bias risk was rated by manipulating the Cochrane risk of bias tool 2.0 (RoB 2). The retrieved investigations were analyzed by utilizing ReviewManager 5.4 and Stata 17.0. The quality of evidence was evaluated adopting the GRADE tool.

**Results:**

A total of 16 clinical RCTs of XBXT in the treatment of CINV were incorporated into the investigation, with a total of 1246 participants. The meta-analysis showed that compared with conventional antiemetic drugs, XBXT and antiemetics improved the vomiting relief efficiency (RR 1.35, 95% confidence interval: 1.25–1.46, *p* < 0.00001), nausea relief efficiency (N = 367, RR 1.23, 95% CI: 1.09–1.38, *p* < 0.00001), and quality of life (RR = 1.37, 95% CI: 1.14–1.65, *p* = 0.0009) and reduced the adverse events (N = 370, RR 0.53, 95% CI: 0.29–0.96, *p* = 0.04). XBXT and DARAs raised eating efficiency compared with DARAs (N = 208, RR 1.30, 95% CI: 1.07–1.57, *p* = 0.007). The data existed as statistically significant, and the publication bias was identified as relatively low from the funnel plot and trim and fill analysis. In addition, sensitivity analysis demonstrated robust outcomes. The quality of evidence for each outcome ranged from moderate to high.

**Conclusion:**

There is some encouraging evidence that XBXT and antiemetics had better therapeutic effects and safety in treating CINV than antiemetic drugs alone. The quality assessment and low publication bias indicated that the overall criterion was scientific. Better research is required to verify the evidence designed with large-scale RCTs and rigorous methods.

**Systematic Review Registration:** https://www.crd.york.ac.uk/PROSPERO/display_record.php?RecordID=281046.

## 1 Introduction

Cancer is a serious disease that endangers human health, and its frequency is expanding year after year (Xia et al., 2022; Siegel et al., 2024). According to the information issued by the International Agency for Research on Cancer (IARC) of the World Health Organization, there will be 28.4 million new cases of cancer worldwide in 2040, an increase of 47% over 2020 (Bray et al., 2024).

As one of the comprehensive treatments for cancer, chemotherapy can cause many uncomfortable reactions while killing cancer cells (Gupta et al., 2021). Chemotherapy-induced nausea and vomiting (CINV) is a frequent adverse effect of chemotherapy, and its incidence is as high as 70%–80% (Piechotta et al., 2021). Due to the degree of vomiting induced by diverse chemotherapeutic drugs, CINV includes acute vomiting and delayed vomiting. Acute vomiting occurs within 24 h after chemotherapy, whereas delayed vomiting occurs after 24 h (Herrstedt et al., 2024). CINV can result in anxiety, depression, and other negative emotions in patients; significantly reduce their quality of life (Farrell et al., 2013); and even develop serious metabolic complications, such as hyponatremia, hypokalemia, and metabolic acidosis, affecting the therapeutic effect (Miao et al., 2017).

The pathogenesis of CINV has not been fully understood, and most scholars believe that it mainly includes the following aspects: chemotherapeutic drugs directly stimulate chromaffin cells in the gastrointestinal tract, which release 5-hydroxytryptamine (5-HT) bound to 5-hydroxytryptamine receptors, producing nerve impulses that act on the vomiting center, leading to CINV (Navari, 2015). Chemotherapeutic drugs and their metabolites cause CINV by stimulating the chemoreceptor trigger zone (CTZ) (Cubeddu, 1992). Sensory and psychiatric factors stimulate the cerebral cortex pathway leading to CINV (Was et al., 2022). Neurotransmitters such as 5-HT_3_, dopamine (DA), substance P (SP), and angiotensin can cause vomiting by stimulating CTZ and vomiting centers (Shankar et al., 2015; Chen et al., 2022).

At present, the drugs for preventing CINV mainly include 5-HT_3_ receptor antagonists, dopamine receptor antagonists, NK-1 receptor antagonists, glucocorticoids, and antihistamines (Rojas et al., 2014; Rojas and Slusher, 2015; Hesketh et al., 2020; Kennedy et al., 2024). The antiemetic mechanisms of different drugs are also different. The most common is to prevent or alleviate nausea and vomiting by inhibiting the chemoreceptor trigger zone (Minami et al., 2003; Smyla et al., 2020). However, these drugs are prone to serious adverse reactions, such as dizziness, constipation, fatigue, and extrapyramidal symptoms (Vardy et al., 2006; Navari and Aapro, 2016).

There is no discussion about CINV in traditional Chinese medicine, but it can be classified into the categories of “vomiting” and “nausea.” Chemotherapeutic drugs also destroy normal human cells when killing cancer cells, which leads to impaired vital energy, viscera dysfunction, spleen dysfunction, and stomach disharmony, inducing a range of gastrointestinal reactions, such as nausea and vomiting. Therefore, the prevention and treatment principle of traditional Chinese medicine (TCM) for CINV is harmonizing the stomach and lowering adverse qi.

Xiao-Ban-Xia-Tang (XBXT) originates from the Treatise on Febrile and Miscellaneous Diseases (Shang Han Za Bing Lun), written by Zhongjing Zhang. XBXT consists of two herbs: *Pinellia ternata* (Banxia) and fresh ginger (Shengjiang). *Pinellia ternata* is the dry tuber of *Pinellia ternata* (Thunb). Breit. However, unprocessed *Pinellia ternata* has toxic effects, with common symptoms including stinging sensations in the throat and mouth and inductions of vomiting and miscarriage. Processed products of *Pinellia ternata* are commonly used in clinical practice (Bai et al., 2022; Peng et al., 2022; Zou et al., 2023). Studies have shown that *Pinellia ternata* contains alkaloids, volatile oil, organic acids, sterols and other chemical components (Oshio et al., 1978; Maki et al., 1987; Niijima et al., 1993; Tomoda et al., 1994; Chen et al., 2003; Jin et al., 2012; Lee et al., 2016), which have antitussive and expectorant, antiemetic, anti-early pregnancy, anti-ulcer, and anti-tumor pharmacological effects (Lu et al., 2012; Chen et al., 2013; Li et al., 2014; Zu et al., 2014). Pinellia alkaloids are the main antiemetic components. Blocking the 5-HT_3_ receptor and NK_1_ receptor may be one of the important mechanisms of Pinellia in preventing CINV.

Fresh ginger is the fresh rhizome of *Zingiber officinale* Roscoe. Ginger contains chemical compounds such as volatile oil, gingerol, flavonoids, and free amino acids (Tao et al., 2009; Baliga et al., 2011; Mao et al., 2019; Li et al., 2021), with antiemetic, anti-inflammatory, antibacterial, antioxidant, antitumor, and other pharmacological effects (Dugasani et al., 2010; Soltani et al., 2017; Nunes et al., 2020; Gao et al., 2024). Ginger, especially its active ingredients, namely, gingerol, 6-gingerol, and 6-shogaol, can inhibit 5-HT_3_ receptors, substance P receptors, and choline receptors to exert an antiemetic effect (Abdel-Aziz et al., 2006; Qian et al., 2010; Kim et al., 2023). In addition, it can regulate vasopressin release, gastrointestinal motility, and gastric emptying rate (Marx et al., 2017; Zhang et al., 2021).

XBXT is mainly used to treat nausea and vomiting caused by various reasons in clinical practice, such as pregnancy vomiting, nervous vomiting, vomiting caused by gastric retention, acute myocardial infarction vomiting, vomiting after chemotherapy, and intractable vomiting (Ji, 2000; Chen and Lu, 2005; Guo et al., 2005; Yu, 2006; Fan, 2011; Li, 2011; Lu and Liu, 2011).

XBXT may prevent CINV by inhibiting 5-HT release and SP synthesis, reducing dopamine content, and blocking the corresponding receptors (Xu and Lian, 2004; Nie and Ma, 2007; Qian et al., 2010; Wang et al., 2010; Qian et al., 2011; Yu et al., 2015a; Yu et al., 2015b; Li et al., 2020; Meng et al., 2020). XBXT can also alleviate gastrointestinal mucositis and delayed gastric emptying caused by chemotherapy drugs (Li et al., 2017; Liu et al., 2017; Du et al., 2018). XBXT combined with 5-hydroxytryptamine-3 receptor antagonists (5-HT3RAs) or dopamine receptor antagonists (DARAs) has a remarkable therapeutic effect in preventing and treating CINV (Ouyang et al., 2002; Guo et al., 2005; Liu and Wang, 2008), but there is not adequate testimony to demonstrate these discoveries. Therefore, this systematic review is the first comprehensive assessment of XBXT in treating CINV, to supply reference for clinical application.

## 2 Methods

The systematic review was manipulated in conformity with the Cochrane Handbook for Systematic Reviews of Interventions and presented complying with the PRISMA (Preferred Reporting Items for Systematic Reviews and Meta-analyses) and PRISMA-CHM 2020 (PRISMA Extension for Chinese Herbal Medicines 2020) guidelines (Page et al., 2021). The PRISMA 2020 checklist is demonstrated in Supplementary Material S1.

### 2.1 Registration and protocol

The protocol of the systematic review was registered in the PROSPERO, and the registration number is CRD42021281046.

### 2.2 Search strategy

The following databases from their inception were systematically searched by two independent investigators for randomized controlled trials (RCTs): the Cochrane Library, PubMed, Embase, Chinese National Knowledge Infrastructure (CNKI), Chinese Scientific Journal Database (VIP), and the Wanfang database. The ongoing and registered trials were retrieved from the Clinicaltrials.gov database and the Chinese Clinical Trial Registry (ChiCTR).

The search strategy used for this updated review was similarly based upon the following terms: “Xiaobanxia Tang,” “Xiaobanxia,” “neoplasm,” “drug therapy, and” “nausea.” The retrieval strategies of several databases are displayed in Supplementary Material S2. Two research workers conducted separate searches of the databases and manual retrieval to search for all relevant research literature. All divergences between assessors were settled by deliberation with the third investigator.

### 2.3 Inclusion and exclusion criteria

#### 2.3.1 Inclusion criteria

(1) Type of studies: only RCTs were included and not restricted by sources or countries. The language of publication was confined to English or Chinese.
(2) Type of participants: grown-up patients were diagnosed with cancer and treated with chemotherapy. The type, pathological type, and stage of cancer were not restricted. Patients had no demographic restrictions such as age, gender, or race.
(3) Type of interventions: intervention measures included XBXT or modified XBXT, not limited by dosage form (decoctions, capsules, pills, or granules), frequency, or dosage. The experimental group can be treated with either XBXT individually or XBXT combined with the control group.
(4) Type of comparisons: the control group can be treated with a placebo or conventional therapy. Conventional therapy involves classic western medicine treatments such as antiemetic drugs.
(5) Types of outcome measures: vomiting and nausea relief efficiency were the primary outcome indicators. The secondary indicators included eating efficiency, adverse reactions, and quality of life.

#### 2.3.2 Exclusion criteria

(1) The literature studies were reviews, case reports, animal studies, or non-RCTs.
(2) Patients have suffered from acute infections, mental disorders, gastrointestinal diseases, or other diseases that may induce nausea and vomiting. Patients received radiation therapy.
(3) Interventions involved TCM treatments other than XBXT, such as acupuncture, moxibustion, or acupoint injection. More than two herbs have been modified in XBXT, or it was not orally administered.
(4) The control group involved treatments other than placebo or conventional therapy.
(5) The research data had obvious errors, questionable authenticity, or deficiency of essential indicators. The information on the investigations was duplicated.

### 2.4 Study selection and data extraction

The retrieved investigations from the databases were sorted into Endnote X9 and screened by two research workers independently. After duplicated studies were removed, all titles and abstracts were reviewed to acknowledge the eligible literature. Then, the full texts were retrieved and evaluated for inclusion. All discrepancies were disposed of by deliberation with a third investigator to reach a consensus.

The information was abstracted and registered in a data-extraction chart by two investigators (Ling Li and Shangmei Jia), respectively. The following elements were collected: fundamental information (title, year, and author), participants (baseline characteristics and sample size), interventions (type, dose, frequency, and procedure of therapies), outcomes (severity and rate of nausea and vomiting and adverse events), outcome indicators, and consequence calculation records of significance.

### 2.5 Risk of bias

The quality of the retrieved references was appraised independently by two investigators (Ling Li and Shangmei Jia) manipulating the Cochrane risk of bias tool 2.0 (RoB 2) (Cumpston et al., 2019). Discrepancies were overcome by a third reviewer (Shasha Shi).

### 2.6 Statistical analysis

RevMan 5.4 software was applied to the meta-analysis of the included documents. The risk ratio (RR) was selected for dichotomous outcomes. For continuous data, mean difference (MD) was used. If the included investigations assessed the outcomes by utilizing multiple scales, the standard mean difference (SMD) would be selected. All the estimates were calculated by 95% confidence intervals (CIs). The chi-square test and *I* ^*2*^ statistics are employed to evaluate the statistical heterogeneity of the retrieved literature. If *I* ^*2*^ > 50%, indicating that there was heterogeneity, the random-effects model was adopted; otherwise, the fixed-effects model was employed. Heterogeneity was processed by subgroup analysis or sensitivity analysis, or only through descriptive analysis to investigate presumable causations from a clinical perspective.

### 2.7 Quality of evidence

The Grading of Recommendations Assessment, Development, and Evaluation (GRADE) tool was adopted to appraise the evidence quality in the review (Izcovich et al., 2023). The quality of evidence was degraded or upgraded by estimating the factors such as the risk of bias, inconsistency, and indirectness. In summary, the quality of evidence was rated as four levels of “high,” “moderate,” “low,” and “very low.”

## 3 Results

### 3.1 Literature search

**FIGURE 1:**
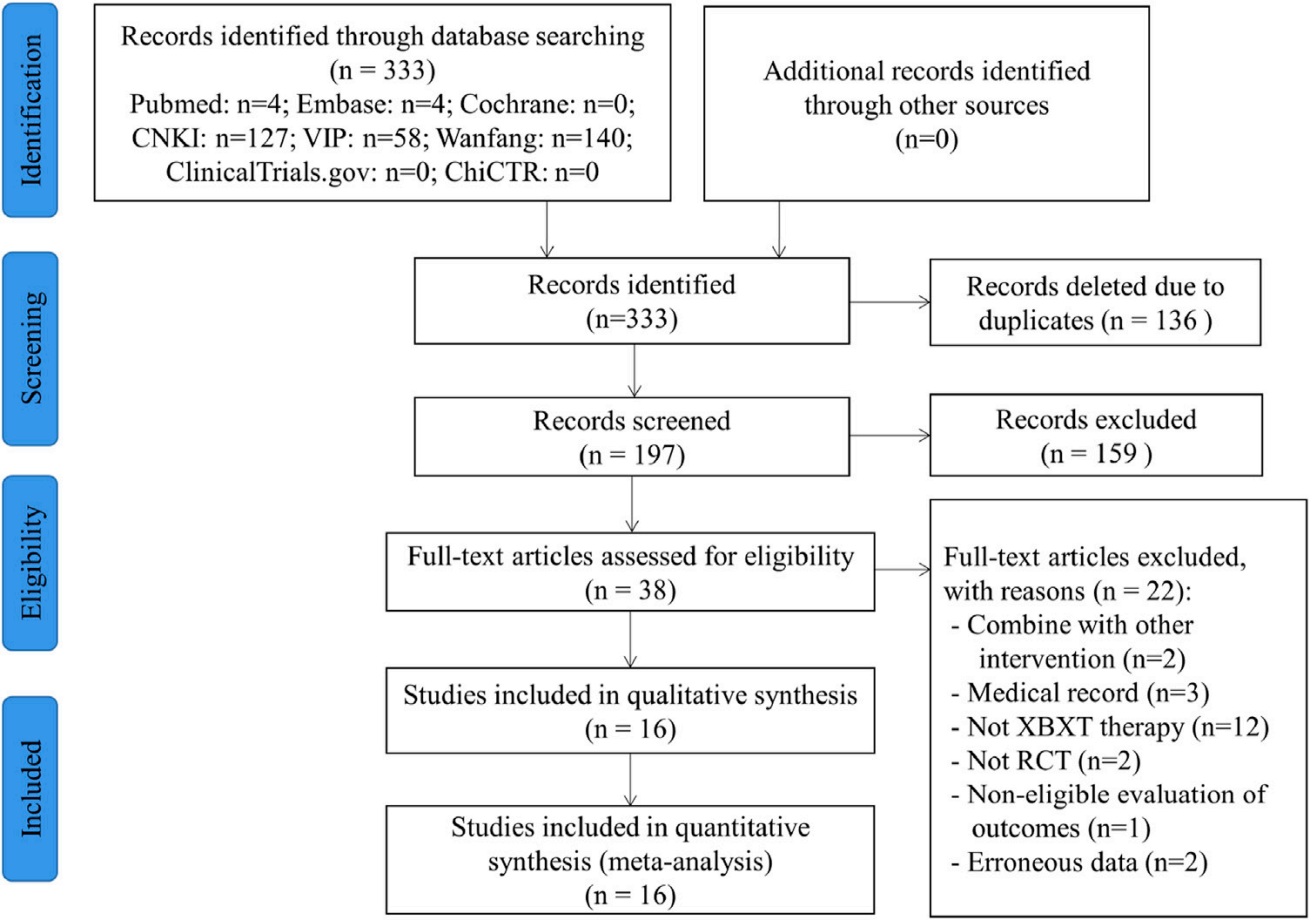
Flow diagram of literature selection.

A total of 333 records were identified through systematic database retrieval and manual retrieval, among which 136 duplicate records were excluded. Through reviewing the titles and abstracts of 197 studies, 159 references were detected as not complying with the demands and were consequently eliminated. The full text of 38 essays was further retrieved and filtered for inclusion. Eventually, 16 writings (Li et al., 1999; Ouyang et al., 2002; Guo et al., 2005; Zhang et al., 2005; Guo, 2008; Liu and Wang, 2008; Liu, 2011; Zheng and Cheng, 2012; Chen et al., 2013; Jiang et al., 2013; Fu et al., 2015; He, 2017; Leng and Li, 2020; Zhang and Wang, 2020; Cui et al., 2021; Tao et al., 2021) fulfilled the criteria for this systematic review. The excluded references and reasons after reading the entire text are exhibited in Supplementary Material S3. The PRISMA flow diagram is represented in Figure 1, which displays the selection process of the entire study.

### 3.2 Characteristics of included studies

The fundamental characteristics of included RCTs are summarized in Table 1 and Table 2. There were 1246 examinees in this investigation, including 628 in the intervention group and 618 in the control group. All the trials were handled in China. All the sick were diagnosed with cancer and received chemotherapy. Fifteen trials (Li et al., 1999; Ouyang et al., 2002; Guo et al., 2005; Zhang et al., 2005; Guo, 2008; Liu and Wang, 2008; Liu, 2011; Zheng and Cheng, 2012; Jiang et al., 2013; Fu et al., 2015; He, 2017; Leng and Li, 2020; Zhang and Wang, 2020; Cui et al., 2021; Tao et al., 2021) compared XBXT and antiemetics with the same individual antiemetics, and one (Chen et al., 2013) compared XBXT with antiemetic drugs. The control group of 11 trials (Ouyang et al., 2002; Guo et al., 2005; Guo, 2008; Liu, 2011; Zheng and Cheng, 2012; Fu et al., 2015; He, 2017; Leng and Li, 2020; Zhang and Wang, 2020; Cui et al., 2021; Tao et al., 2021) utilized 5-HT_3_ receptor antagonists as antiemetics; in addition, seven studies (Li et al., 1999; Zhang et al., 2005; Liu and Wang, 2008; Chen et al., 2013; Jiang et al., 2013; Fu et al., 2015; Zhang and Wang, 2020) adopted dopamine receptor antagonists as antiemetics, of which two studies (Fu et al., 2015; Zhang and Wang, 2020) utilized ondansetron and metoclopramide. Among the significant outcome indicators, 13 studies (Li et al., 1999; Ouyang et al., 2002; Guo et al., 2005; Zhang et al., 2005; Guo, 2008; Liu and Wang, 2008; Zheng and Cheng, 2012; Jiang et al., 2013; Fu et al., 2015; He, 2017; Leng and Li, 2020; Zhang and Wang, 2020; Tao et al., 2021) reported overall efficiency of CINV, and four studies (Guo et al., 2005; Liu and Wang, 2008; Leng and Li, 2020; Cui et al., 2021) reported adverse reaction rates.

**TABLE 1:** Characteristics of included trials.

Study ID	Country	Male/female ratio (T; C)	Average age (years)(T/C)	Sample size (T/C)	Intervention regime		Treatment duration	Outcomes
					T	C		
Chen et al. (2013)	China	25/21; 22/23	71.2 ± 7.76 (59–79)	91 (46/45)	XBXT	Metoclopramide	3 days	① and ⑦
Cui et al. (2021)	China	Not reported	48 ± 8/49 ± 7	128 (64/64)	XBXT + granisetron	Granisetron	7 days	①, ②, ④, and ⑨
Fu et al. (2015)	China	17/13; 20/10	62.50 ± 3.18/63.83 ± 2.35	60 (30/30)	XBXT + metoclopramide	Ondansetron + metoclopramide	During chemotherapy	①, ③, and ⑤
Guo (2008)	China	34/28	31–76	62 (32/30)	XBXT + azasetron	Azasetron	11 days	①, ②, ③, and ④
Guo et al. (2005)	China	62/38	18–80	100 (50/50)	XBXT + granisetron	Granisetron	7 days	①, ③, and ⑨
He (2017)	China	18/12; 17/13	65.3 ± 2.1 (55–76)/66.1 ± 2.3 (52–77)	60 (30/30)	XBXT + moxibustion + tropisetron	Tropisetron	9 days	① and ③
Jiang et al. (2013)	China	20/25	41 (20–72)	45 (20/25)	XBXT + Maxolon	Maxolon	9 days	①, ③, and ⑧
Leng and Li (2020)	China	29/21; 26/24	64.83 ± 3.98 (50–78)/64.87 ± 4.02 (52–78)	100 (50/50)	XBXT + ondansetron hydrochloride	Ondansetron hydrochloride	5 days	①, ②, ③, and ⑨
Li et al. (1999)	China	72/49	43 (17–72)	121 (64/57)	XBXT + maxolon	Maxolon	10 days	①, ②, ③, and ⑧
Liu (2011)	China	20/20	30–60	40 (20/20)	XBXT + granisetron	Granisetron	5 days	⑥
Liu and Wang (2008)	China	25/17	46 (31–73)	42 (21/21)	XBXT + metoclopramide	Metoclopramide	6 days	①, ②, ③, ⑧, and ⑨
Ouyang et al. (2002)	China	50/23; 43/29	52.5 (17–78)/50.3 (20–75)	145 (73/72)	XBXT + ondansetron	Ondansetron	From 2 days before to 2 days after chemotherapy	① and ③
Tao et al. (2021)	China	0/50; 0/50	54.30 ± 8.98/53.26 ± 9.64	100 (50/50)	XBXT + ondansetron	Ondansetron	7 days	①, ②, ③, and ⑧
Zhang et al. (2005)	China	24/18	41 (32–67)	42 (23/19)	XBXT + maxolon	Maxolon	10 days	①, ②, and ③
Zhang and Wang (2020)	China	19/16; 20/15	63 ± 4/62.5 ± 3.5	70 (35/35)	XBXT + metoclopramide	Ondansetron + metoclopramide	5 days	①, ③, and ⑤
Zheng and Cheng (2012)	China	11/9; 13/7	34.25 ± 9.11 (16–55)/35.45 ± 10.02 (16–55)	40 (20/20)	XBXT + ondansetron	Ondansetron	7 days	① and ③

① vomiting frequency; ② nausea frequency; ③ overall efficiency of CINV; ④ complete control rate; ⑤ quality of life; ⑥ grading of gastrointestinal symptoms; ⑦ safety evaluation; ⑧ appetite; ⑨ incidence of adverse reactions.

**TABLE 2:** Cancer type and components of prescriptions.

Study ID	Cancer type	Chemotherapy regimen	Medical institution	Component(g)
Chen et al. (2013)	Various cancers	Platinum-based combination chemotherapy	Nanjing Hospital of TCM, Nangjing, China	Pinellia Rhizome (Banxia, Rhizoma Pinelliae) 10g, fresh ginger Rhizome (Shengjiang, Zingiber officinale Roscoe) 20g, and Tuckahoe (Fu Ling, Poria 12 g
Cui et al. (2021)	Breast cancer	AC/EC (doxorubicin + cyclophosphamide/epirubicin + cyclophosphamide)	Chinese PLA General Hospital, Beijing, China	Pinellia Rhizome (Banxia, Rhizoma Pinelliae) 10 g and fresh ginger Rhizome (Shengjiang, Zingiber officinale Roscoe) 10 g
Fu et al. (2015)	Gastric cancer	Paclitaxel and cisplatin	First Affiliated Hospital of Chinese PLA General Hospital, Beijing, China	Pinellia Rhizome (Banxia, Rhizoma Pinelliae) 9 g and fresh ginger Rhizome (Shengjiang, Zingiber officinale Roscoe) 15 g
Guo (2008)	Various cancers	Cisplatin-based chemotherapy	Jiaozuo Second People‘s Hospital of Henan Province, Jiaozuo, China	Pinellia Rhizome (Banxia, Rhizoma Pinelliae) 15g, fresh ginger Rhizome (Shengjiang, Zingiber officinale Roscoe) 15g, and Tuckahoe (Fu Ling, Poria 30g
Guo et al. (2005)	Various cancers	Cisplatin-based chemotherapy	Nanyang Medical College Affiliated Hospital, Nanyang, China	Pinellia Rhizome (Banxia, Rhizoma Pinelliae) 15g and fresh ginger Rhizome (Sheng Jiang, Zingiber officinale Roscoe) 20g
He (2017)	Lung cancer	Paclitaxel and cisplatin	Jiaxing Hospital of TCM, Jiaxing, China	Pinellia Rhizome (Banxia, Rhizoma Pinelliae) 20g and fresh ginger Rhizome (Shengjiang, Zingiber officinale Roscoe) 25g
Jiang et al. (2013)	Various cancers	Cisplatin-based chemotherapy	Ningbo Hospital of TCM, Ningbo, China	Pinellia Rhizome (Banxia, Rhizoma Pinelliae) 20g and fresh ginger Rhizome (Sheng Jiang, Zingiber officinale Roscoe) 25g
Leng and Li (2020)	Gastric cancer	TP (paclitaxel and platinum)	The Second Affiliated Hospital to Xinjiang Medical University, Urumqi, China	Pinellia Rhizome (Banxia, Rhizoma Pinelliae) 10g and fresh ginger Rhizome (Shengjiang, Zingiber officinale Roscoe) 5g
Li et al. (1999)	Various cancers	Cisplatin-based chemotherapy	Affiliated Hospital of North Sichuan Medical College, Nanchong, China	Pinellia Rhizome (Banxia, Rhizoma Pinelliae) 15g and fresh ginger Rhizome (Shengjiang, Zingiber officinale Roscoe) 20g
Liu (2011)	Lung cancer	EP (cisplatin + etoposide)	TCM tumor Hospital of Henan Province Zhengzhou Huiji District, Zhengzhou, China	Pinellia Rhizome (Banxia, Rhizoma Pinelliae) 30g and fresh ginger Rhizome (Shengjiang, Zingiber officinale Roscoe) 30g
Liu and Wang (2008)	Various cancers	Cisplatin-based chemotherapy	Cancer Hospital of Affiliated Hospital of Zunyi Medical College, Zunyi, China	Pinellia Rhizome (Banxia, Rhizoma Pinelliae) 50g, fresh ginger Rhizome (Shengjiang, Zingiber officinale Roscoe) 50g, and Tuckahoe (Fu Ling, Poria) 50g
Ouyang et al. (2002)	Various cancers	Mixed. Multiple regimens of varying emetogenicity	Fuzhou General Hospital of Nanjing Military Region, Fuzhou, China	Pinellia Rhizome (Banxia, Rhizoma Pinelliae) 15g and fresh ginger Rhizome (Shengjiang, Zingiber officinale Roscoe) 20g
Tao et al. (2021)	Gynecological cancer	Paclitaxel + carboplatin, paclitaxel + nedaplatin, and paclitaxel + bevacizumab	Changzhou Maternal and Child Health Hospital Affiliated to Nanjing Medical University, Changzhou, China	Pinellia Rhizome (Banxia, Rhizoma Pinelliae) 18g and fresh ginger Rhizome (Shengjiang, Zingiber officinale Roscoe) 9g
Zhang et al. (2005)	Lung cancer	PE (cisplatin + etoposide)	Affiliated Hospital of Henan Academy of TCM, Zhengzhou, China	Pinellia Rhizome (Banxia, Rhizoma Pinelliae) 20g, fresh ginger Rhizome (Shengjiang, Zingiber officinale Roscoe) 15g, and Tuckahoe (Fu Ling, Poria) 30g
Zhang and Wang (2020)	Gastric cancer	Paclitaxel + cisplatin	Xing ‘an Street Health Center of Anqiu City, Weifang, China	Pinellia Rhizome (Banxia, Rhizoma Pinelliae) 9g and fresh ginger Rhizome (Shengjiang, Zingiber officinale Roscoe) 15g
Zheng and Cheng (2012)	Acute myeloid leukemia	Mixed	The Second Affiliated Hospital of Henan College of TCM, Zhengzhou, China	Pinellia Rhizome (Banxia, Rhizoma Pinelliae) 18g and fresh ginger Rhizome (Shengjiang, Zingiber officinale Roscoe) 15g

### 3.3 Risk of bias assessment of the included studies

**FIGURE 2:**
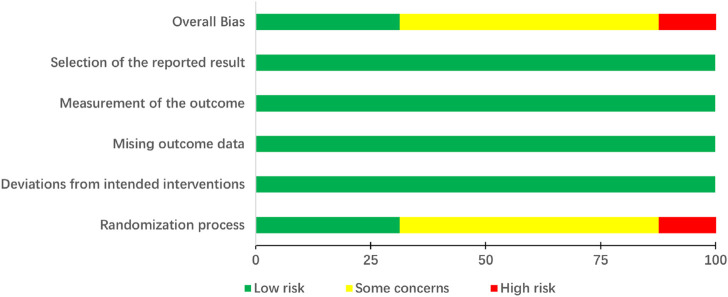
Risk of bias graph (RoB 2).

**FIGURE 3:**
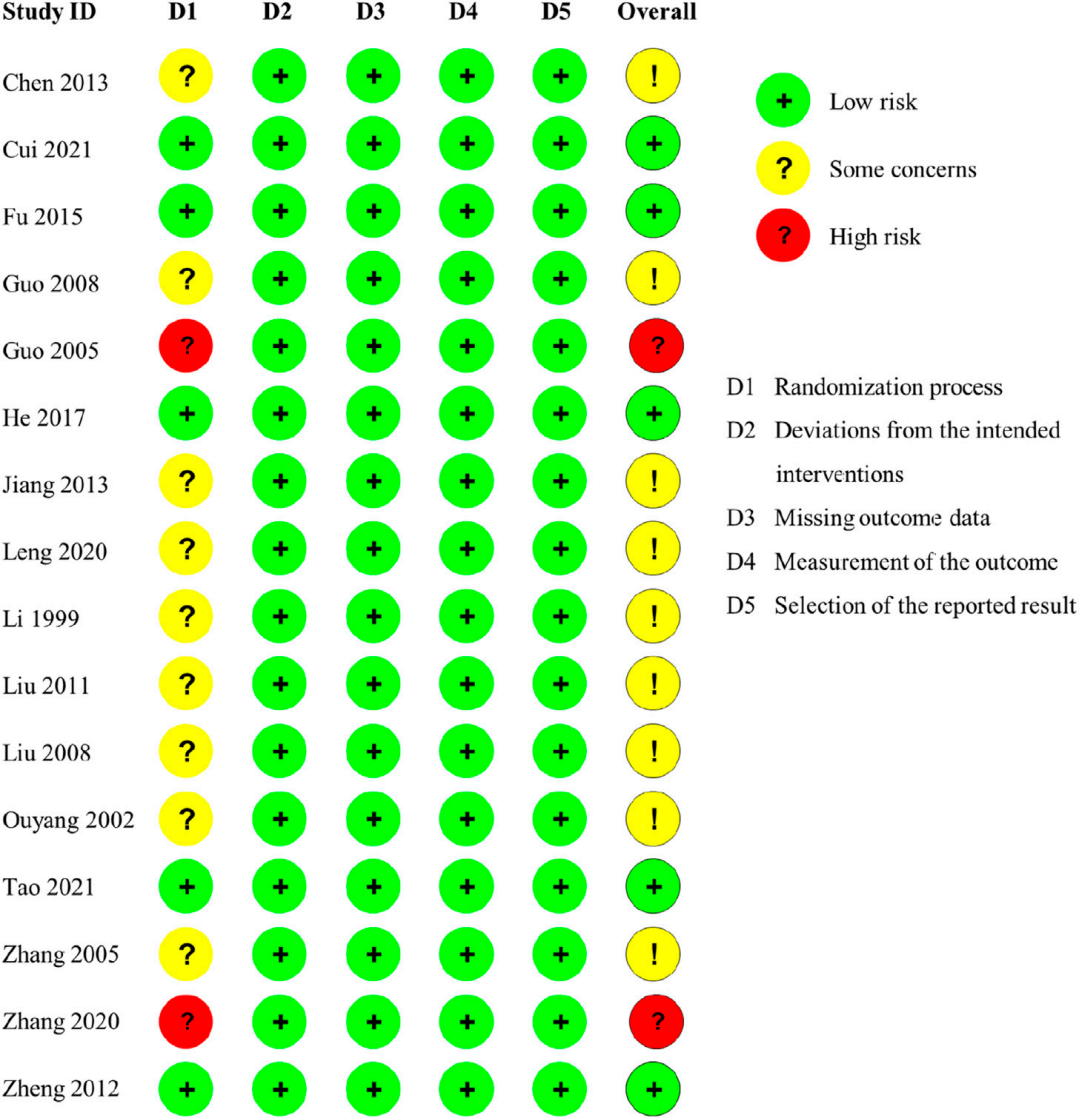
Risk of bias summary (RoB 2).

The assessment outcomes of the included 16 investigations using the RoB 2.0 tool are exhibited in Figure 2 and Figure 3. Randomization was mentioned in all studies. Four studies (Zheng and Cheng, 2012; Fu et al., 2015; Cui et al., 2021; Tao et al., 2021) mentioned the generation of random sequences through random number tables. Only one investigation (He, 2017) referred to the method of drawing lots and allocation concealment. Consequently, these five trials were labeled as low risk in the randomization process. One study (Zhang and Wang, 2020) was grouped by the odd–even number method, and one study (Guo et al., 2005) was grouped according to the date of admission. Both studies were identified as high risk owing to improper randomization approaches. Other research studies have not reported measures for generating random sequences; thus, these research studies have been labeled as unclear risk. In all trials, although participants were aware of the intervention measures, it possibly did not affect the outcomes. Moreover, the analysis means and outcome of measurement in all included RCTs were appropriate. The outcomes of all investigations were impartial, and there were no missing data. Therefore, the interventions, missing data, measurement, and reported results of all trials were marked as low risk. In summary, the majority of research studies had either low or unclear risk of bias, with only two studies (Guo et al., 2005; Zhang and Wang, 2020) having high risk of overall bias.

### 3.4 Meta-analysis outcome

#### 3.4.1 Vomiting relief efficiency

**FIGURE 4:**
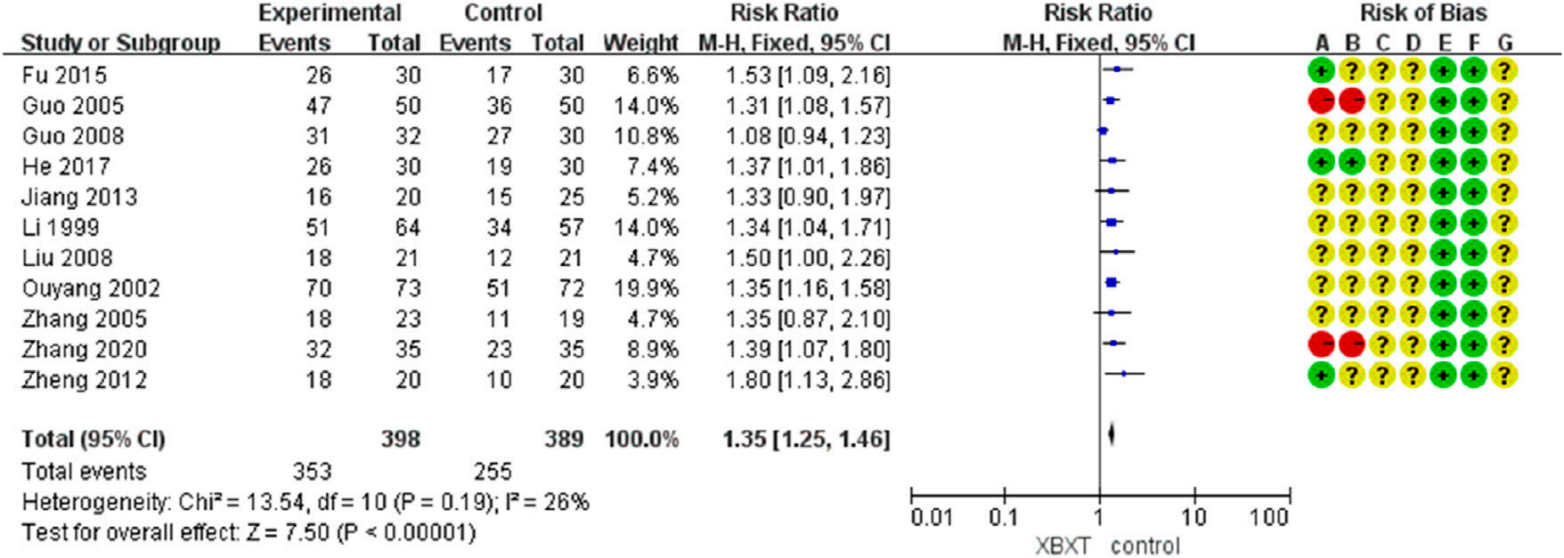
Forest plot of vomiting relief efficiency.

A total of 11 studies (Li et al., 1999; Ouyang et al., 2002; Guo et al., 2005; Zhang et al., 2005; Guo, 2008; Liu and Wang, 2008; Zheng and Cheng, 2012; Jiang et al., 2013; Fu et al., 2015; He, 2017; Zhang and Wang, 2020) were evaluated to estimate the vomiting relief efficiency. The results of heterogeneity showed that *p* = 0.19 and *I* ^2^ = 26%, which indicated that the research data were homogeneous. The fixed-effects model was selected, and the analysis results demonstrated that the records showed statistical significance in the effective rate of vomiting relief (N = 787, RR 1.35, 95% CI: 1.25–1.46, *p* < 0.00001), as shown in Figure 4. Sensitivity analysis manifested that there were few differences in the pooled effect size estimates and the outcomes were robust (Figure 14A, Supplementary Material S6.1).

**FIGURE 5:**
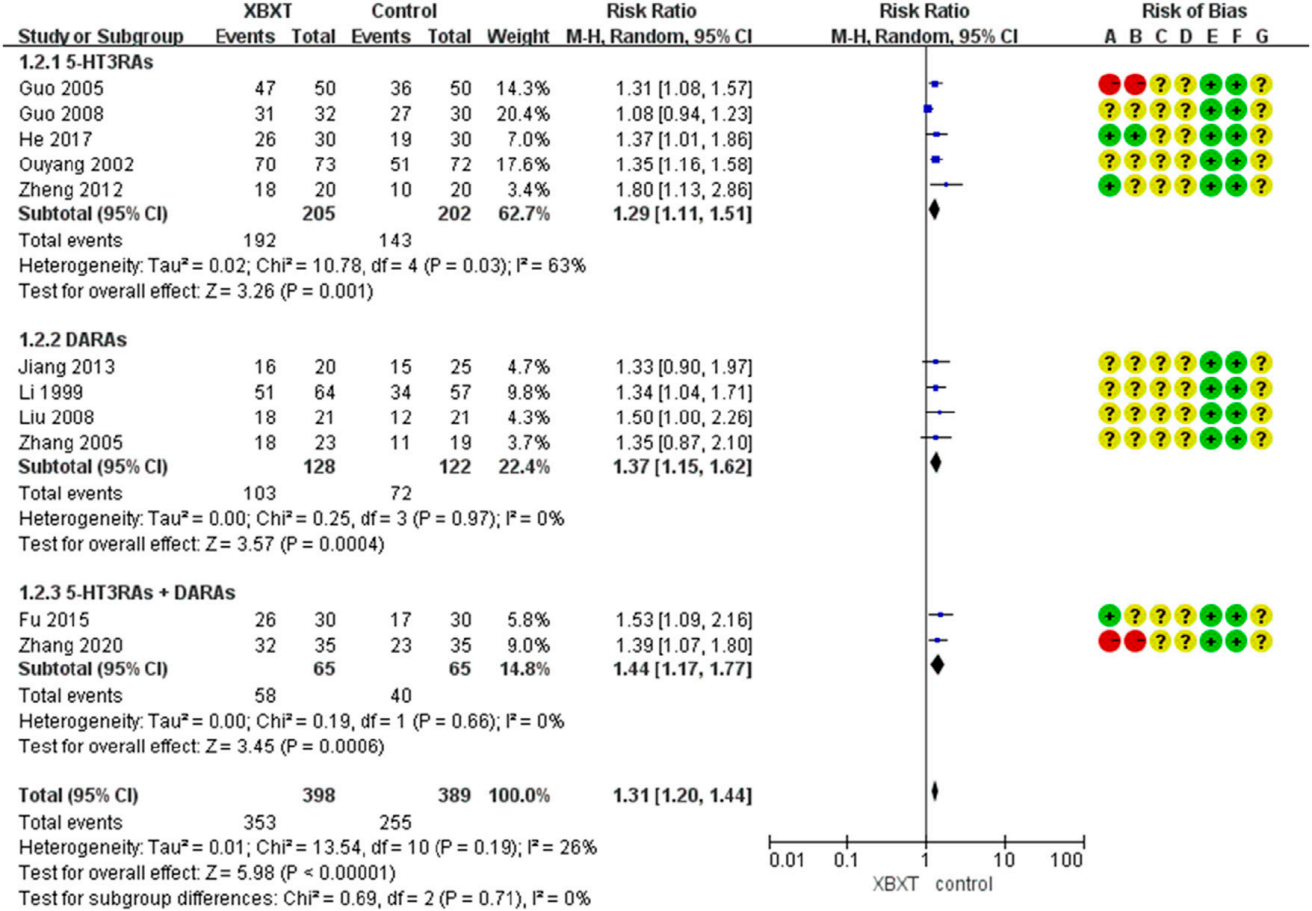
Forest plot of vomiting relief efficiency in various antiemetics.

Subgroup analysis manifested that whether the control group was treated with 5-HT3RAs (N = 407, RR 1.29, 95% CI: 1.11–1.51, *p* = 0.001), DARAs (N = 250, RR 1.37, 95% CI: 1.15–1.62, *p* = 0.0004), or 5-HT3RAs + DARAs (N = 130, RR 1.4495% CI: 1.17–1.77, *p* = 0.0006), XBXT could improve vomiting relief efficiency (Figure 5).

**FIGURE 6:**
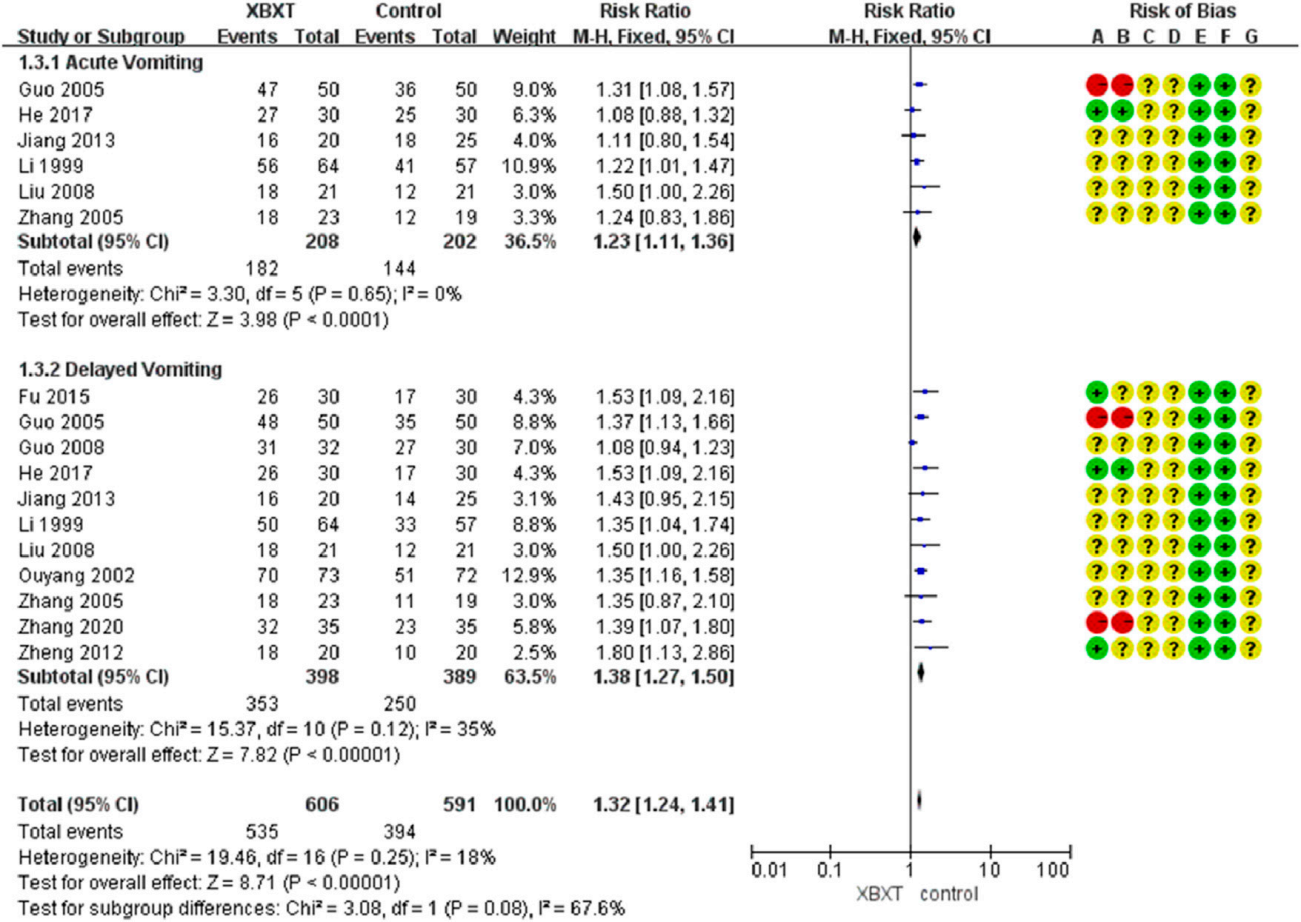
Forest plot of various vomiting patterns.

Based upon the subgroup analysis of vomiting patterns, XBXT combined with antiemetics prominently ameliorated acute vomiting (N = 410, RR 1.23, 95% CI: 1.11–1.36, *p* < 0.0001) (Li et al., 1999; Guo et al., 2005; Zhang et al., 2005; Liu and Wang, 2008; Jiang et al., 2013; He, 2017) and delayed vomiting (N = 787, RR 1.38, 95% CI: 1.27–1.50, *p* < 0.00001) (Li et al., 1999; Ouyang et al., 2002; Guo et al., 2005; Zhang et al., 2005; Guo, 2008; Liu and Wang, 2008; Zheng and Cheng, 2012; Jiang et al., 2013; Fu et al., 2015; He, 2017; Zhang and Wang, 2020) compared with antiemetic drugs (Figure 6). No measurable heterogeneity was identified in the investigation (*I* ^2^ = 18%). Sensitivity analysis of both vomiting patterns displayed similar pooled effect size estimates and stable results (Figures 14B,C, Supplementary Materials S6.2, S6.3).

#### 3.4.2 Publication bias

**FIGURE 7:**
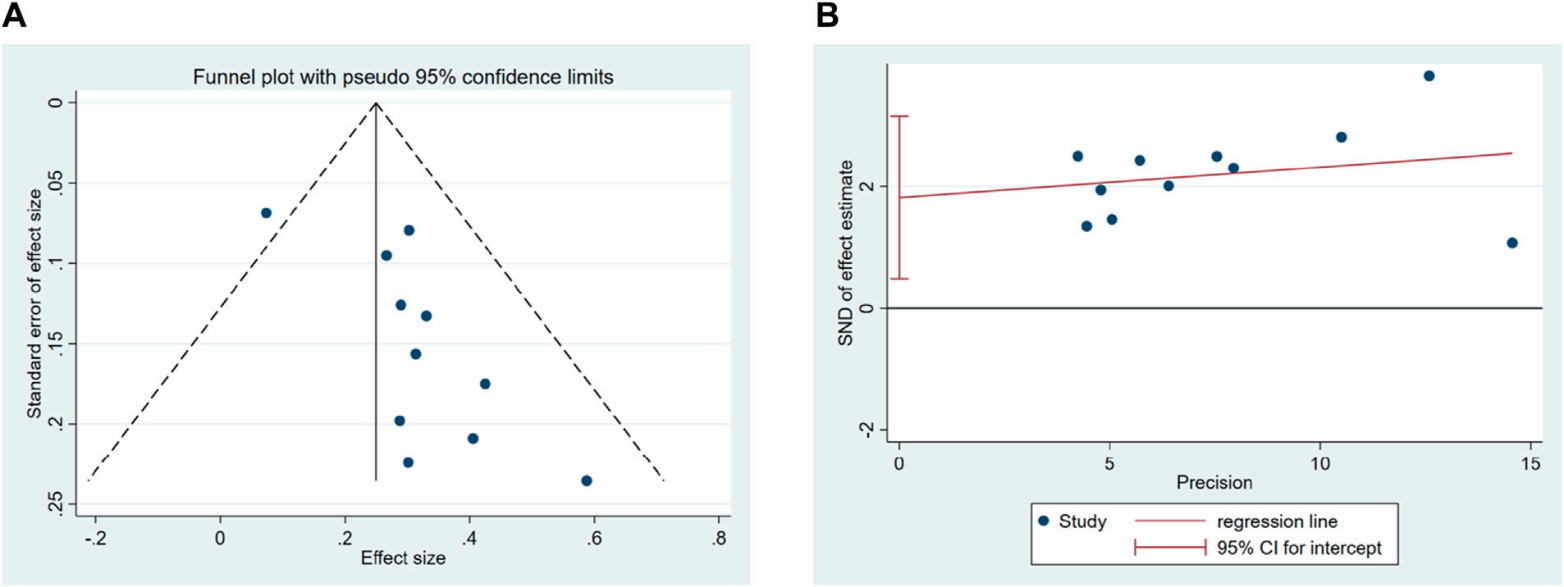
Publication bias about vomiting relief efficiency. **(A)** Funnel plot and **(B)** Egger’s test.

Stata 17.0 was used for the funnel plot to analyze the bias of the included studies. The results are displayed in Figure 7A. It can be identified from the funnel plot that the distribution of the included studies was relatively concentrated; however, the figure was slightly asymmetric. It showed that there was still bias among the included research, but the deviations may not be especially noticeable.

The analysis consequence manifested that the graph was asymmetric, with most of the data distributed on the right side of the funnel plot, which suggested that there was the possibility of publication bias. Egger’s test demonstrated potential publication bias in the investigations of vomiting relief efficiency (*p* = 0.013) (Figure 7B, Supplementary Material S4.1). Therefore, it was required to adopt the trim and fill analysis. Through the results of the trim and fill analysis, it was identified that there was no distinct variation in the estimated value of the pooled effect size, indicating that the impact of publication bias was not evident and the outcomes were quite robust (Supplementary Material S5).

#### 3.4.3 Nausea relief efficiency

**FIGURE 8:**
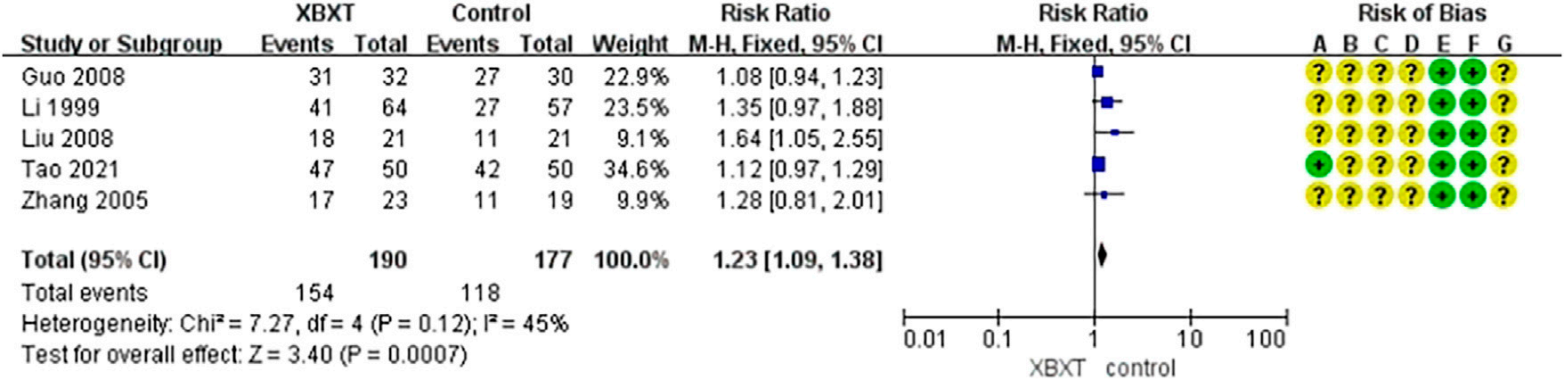
Forest plot of nausea relief efficiency.

A total of five studies (Li et al., 1999; Zhang et al., 2005; Guo, 2008; Liu and Wang, 2008; Tao et al., 2021) were evaluated to estimate the nausea relief efficiency. The outcomes of the heterogeneity test identified that *p* = 0.12 and *I* ^2^ = 45%, proving that the included research data were homogeneous. The fixed-effects model was adopted, and the results of meta-analysis revealed that the data existed statistically significant in the efficiency of nausea relief (N = 367, RR 1.23, 95% CI: 1.09–1.38, *p* = 0.0007) (Figure 8). Sensitivity analysis revealed slight discrepancies in pooled effect size estimates and robust outcomes (Figure 14D, Supplementary Material S6.4).

**FIGURE 9:**
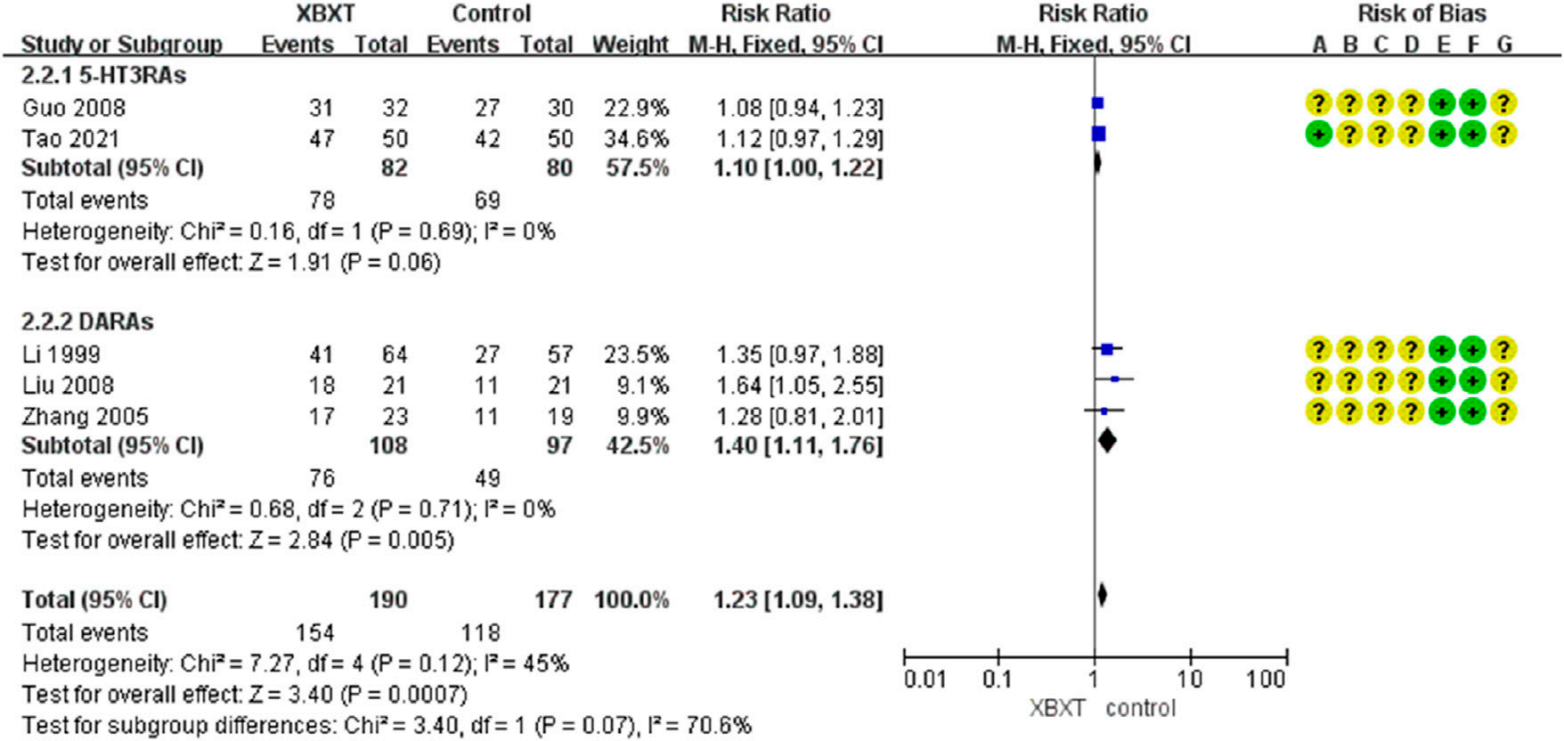
Forest plot of nausea relief efficiency in various antiemetics.

Subgroup analysis based on distinct antiemetics reflected a reduction in heterogeneity within each subgroup (*I* ^2^ = 0%, *I* ^2^ = 0%). Nevertheless, compared with 5-HT3RAs, the combination of XBXT and 5-HT3RAs did not reveal a remarkable statistical significance in lowering the nausea relief rate (N = 162, RR 1.10, 95% CI: 1.00–1.22, *p* = 0.06). Compared with DARAs, XBXT combined with DARAs significantly lessened nausea relief efficiency (N = 205, RR 1.40, 95% CI: 1.11–1.76, *p* = 0.005) (Figure 9).

#### 3.4.4 Eating efficiency

**FIGURE 10:**
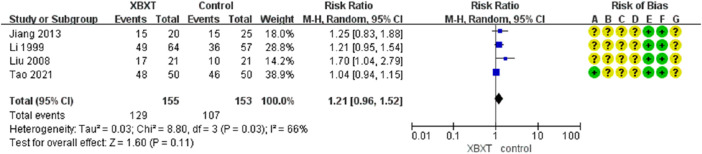
Forest plot of eating efficiency.

A total of four studies (Li et al., 1999; Liu and Wang, 2008; Jiang et al., 2013; Tao et al., 2021) were evaluated to estimate the eating efficiency. Three trials were treated with DARAs, whereas one trial was treated with 5-HT3RA. The random-effects model was adopted due to the heterogeneity of *p* = 0.03 and *I* ^2^ = 66%. However, the results of meta-analysis demonstrated that the data were not statistically significant in terms of the eating rate (N = 308, RR 1.21, 95% CI: 0.96–1.52, *p* = 0.11) (Figure 10).

**FIGURE 11:**
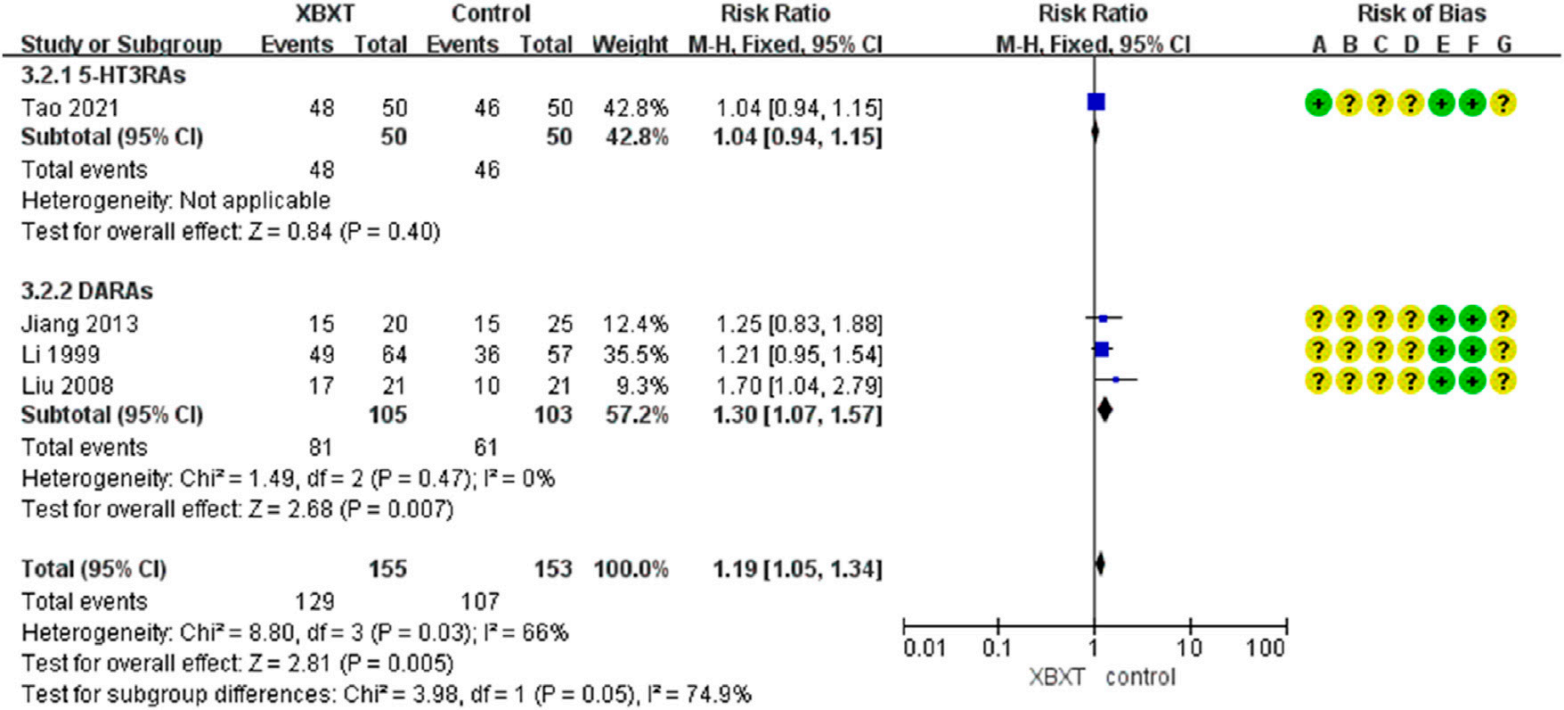
Forest plot of eating efficiency in various antiemetics.

According to the subgroup analysis of distinct antiemetic drugs, heterogeneity has been significantly lowered (*p* = 0.47, *I* ^2^ = 0%) (Figure 11). Consequently, different antiemetic drugs may be heterogeneous sources for eating efficiency. Sensitivity analysis indicated a relatively high level of sensitivity in the study by Tao et al. (2021) (Figure 14E, Supplementary Material S6.5). After removing the study of Tao et al. (2021), XBXT and DARAs promoted eating efficiency compared with DARAs, and the results were statistically significant (N = 208, RR 1.30, 95% CI: 1.07–1.57, *p* = 0.007) (Supplementary Material S6.6). It illustrated that the study was the major origin of heterogeneity.

#### 3.4.5 Adverse events

**FIGURE 12:**
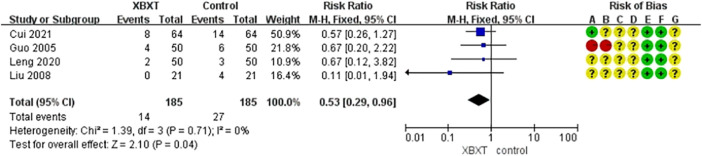
Forest plot of adverse events.

A total of four studies (Guo et al., 2005; Liu and Wang, 2008; Leng and Li, 2020; Cui et al., 2021) were appraised for adverse events. The control groups of three investigations were treated with 5-HT3RAs, whereas one investigation was treated with DARAs. The analysis outcomes of adverse reactions are presented in Figure 10. The homogeneity of the data was fairly good (*p* = 0.71, *I* ^2^ = 0%). Accordingly, the fixed-effects model was employed for statistical analysis. The adverse effect rate of XBXT in treating CINV was lower than that obtained with antiemetics, and the difference existed statistically significant (N = 370, RR 0.53, 95% CI: 0.29–0.96, *p* = 0.04) (Figure 12). Sensitivity analysis demonstrated similar pooled effect size estimates and stable outcomes (Figure 14F, Supplementary Material S6.7).

#### 3.4.6 Quality of life

**FIGURE 13:**
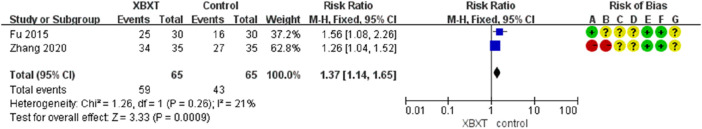
Forest plot of the quality of life.

**FIGURE 14:**
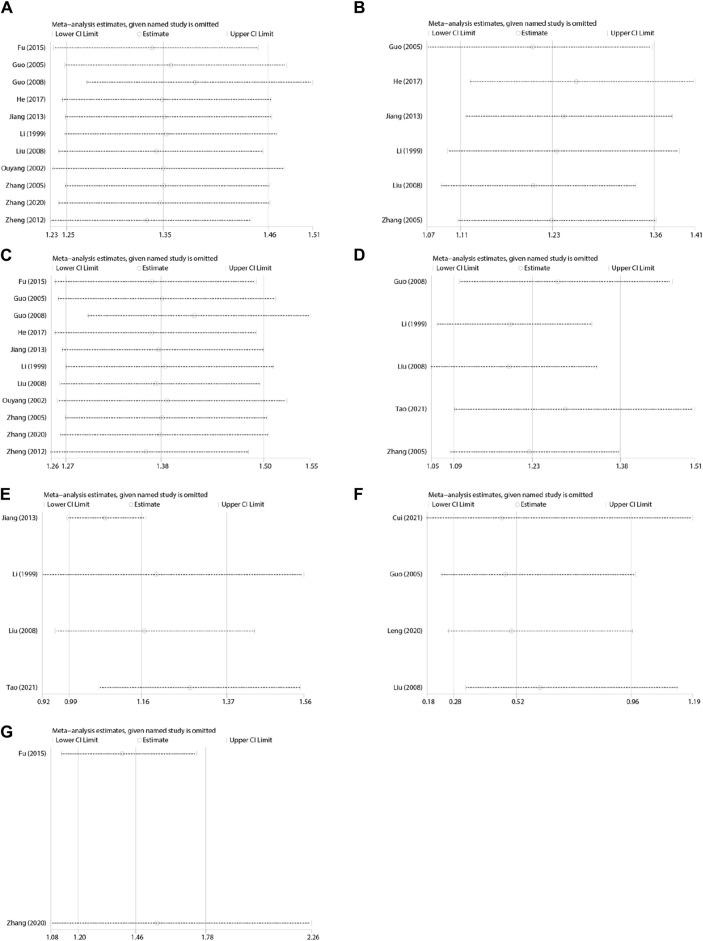
Sensitivity analyses. **(A)** Vomiting relief efficiency, **(B)** acute vomiting, **(C)** delayed vomiting, **(D)** nausea relief efficiency, **(E)** eating efficiency, **(F)** adverse events, and **(G)** quality of life.

A total of two studies (Fu et al., 2015; Zhang and Wang, 2020) were evaluated to estimate the quality of life. Compared with individual antiemetic drugs, XBXT combined with antiemetics markedly upgraded the quality of life (RR = 1.37, 95% CI: 1.14–1.65, *p* = 0.0009) (Figure 13). A fixed-effects model was employed owing to the homogeneity (*p* = 0.26, *I* ^2^ = 21%). Sensitivity analysis displayed few distinctions in the pooled effect size estimates and robust results (Figure 14G, Supplementary Material S6.8).

### 3.5 Grade of the evidence quality

The GRADE method was utilized to estimate the efficacy of XBXT on CINV (Table 3). The evidence quality was moderate in terms of vomiting relief efficiency, delayed vomiting, adverse events, and quality of life. The risk of bias was determined to be serious, leading to a downgrade of the evidence level. Because there were high-risk selection biases, the research studies had unclear risk of performance and detection biases. The evidence quality for eating efficiency was moderate. The imprecision was identified as serious due to the overlap of 95% confidence intervals. The evidence quality was high in terms of acute vomiting and nausea relief efficiency, as the quality assessments were not serious.

**TABLE 3:** GRADE evidence profile.

Quality assessment							No. of patients		Effect		Quality	Importance
No. of studies	Design	Risk of bias	Inconsistency	Indirectness	Imprecision	Other considerations	Clinical efficacy	Control	Relative (95% CI)	Absolute		
**Vomiting relief efficiency**												
11	Randomized trials	Serious^a^	No serious inconsistency	No serious indirectness	No serious imprecision	None	353/398 (88.7%)	255/389 (65.6%)	RR 1.35 (1.25–1.46)	-	⊕⊕⊕Ο MODERATE	CRITICAL
**Acute vomiting**												
6	Randomized trials	No serious risk of bias	No serious inconsistency	No serious indirectness	No serious imprecision	None	182/208 (87.5%)	144/202 (71.3%)	RR 1.23 (1.11–1.36)	-	⊕⊕⊕⊕ HIGH	CRITICAL
**Delayed vomiting**												
11	Randomized trials	Serious^a^	No serious inconsistency	No serious indirectness	No serious imprecision	None	353/398 (88.7%)	250/389 (64.3%)	RR 1.38 (1.27–1.5)	-	⊕⊕⊕Ο MODERATE	CRITICAL
**Nausea relief efficiency**												
5	Randomized trials	No serious risk of bias	No serious inconsistency	No serious indirectness	No serious imprecision	None	154/190 (81.1%)	118/177 (66.7%)	RR 1.23 (1.09–1.38)	-	⊕⊕⊕⊕ High	Critical
**Eating efficiency**												
4	Randomized trials	No serious risk of bias	No serious inconsistency	No serious indirectness	Serious^b^	None	129/155 (83.2%)	107/153 (69.9%)	RR 1.21 (0.96–1.52)	-	⊕⊕⊕Ο Moderate	Critical
**Adverse events**												
4	Randomized trials	Serious^a^	No serious inconsistency	No serious indirectness	No serious imprecision	None	14/185 (7.6%)	27/185 (14.6%)	RR 0.53 (0.29–0.96)	-	⊕⊕⊕Ο Moderate	Critical
**Quality of life**												
2	Randomized trials	Serious^a^	No serious inconsistency	No serious indirectness	No serious imprecision	None	59/65 (90.8%)	43/65 (66.2%)	RR 1.37 (1.14–1.65)	-	⊕⊕⊕Ο Moderate	Critical

^a^ There were high risk of selection biases. All studies had unclear risk of performance and detection biases.

^b^ The 95% confidence interval overlapped with no effect.

## 4 Discussion

CINV is a prevalent adverse symptom after chemotherapy with anti-tumor drugs. Antiemetic drugs have been investigated targeting specific pathways involved in CINV, which can induce impairment to the nerve, digestion, and immunity (Hesketh et al., 2017; Aogi et al., 2021). The traditional Chinese medicine (TCM) exerts a significant influence in the treatment of CINV due to its therapeutic effect, mild toxicity, and ability to alleviate adverse reactions of chemical drugs. Therefore, the combination of TCM and western medicine in treating CINV has become one of the research hotspots. The traditional Chinese medicine prescription XBXT has the characteristics of multi-target, wide curative effect, and small side effects, which makes up for the deficiency of antiemetic drugs at present.

The Chinese herbal formula, XBXT, is derived from the Synopsis of Golden Chamber written by Zhongjing Zhang during the Han Dynasty, which can downbear counterflow and check vomiting. XBXT consists of *Pinellia ternata* (Banxia) and fresh ginger (Shengjiang) that has been utilized for the treatment of vomiting for 1800 years in China. CINV can be quantified by cisplatin-induced augment in kaolin consumption (pica) (Takeda et al., 1993). Rat experiments have demonstrated the antiemetic function of XBXT on CINV in the cisplatin-induced pica model. The function of XBXT is interrelated to the suppression of central or peripheral growth of obestatin, or the levels of cholecystokinin (CCK) and calcitonin gene-related peptide (CGRP) in blood (Qian et al., 2011). XBXT could treat CINV by reducing the content of substance P, the expression of the NK1 receptor, and the level of peripheral and central tyrosine hydroxylase (TH), and by inhibiting the synthesis of dopamine in cisplatin-induced pica rats (Qian et al., 2010; Yu et al., 2015a; Yu et al., 2015b). XBXT can regulate multiple inflammation-related signaling pathways, restraining the activation of NLRP3 inflammasome, the overexpression of pro-inflammation cytokines, and the synthesis of 5-HT (Li et al., 2020; Meng et al., 2020). XBXT can restrain the activation of the ROS/JNK/Bax signaling pathway, decrease GSDME-mediated pyroptosis, and alleviate gastrointestinal inflammation (Liao et al., 2024). In addition, XBXT may activate the AMPK-Nrf2 signaling pathway and reinstate cisplatin-induced PINK1/Parkin-mediated mitochondrial autophagy defects (Zhao et al., 2024).

This is the updated systematic review and meta-analysis investigating the effectiveness of XBXT compared with antiemetics for the prophylaxis of CINV. A total of 16 RCTs were systematically analyzed to estimate the efficacy and safety of XBXT in treating CINV. A total of 16 RCTs involving 1246 subjects were included, all of which were conducted in China. The investigation manifested that XBXT combined with antiemetics was superior to antiemetic drugs in terms of vomiting relief efficiency, nausea relief efficiency, eating efficiency, and quality of life, and the outcomes were statistically significant. Eleven studies (Li et al., 1999; Ouyang et al., 2002; Guo et al., 2005; Zhang et al., 2005; Guo, 2008; Liu and Wang, 2008; Zheng and Cheng, 2012; Jiang et al., 2013; Fu et al., 2015; He, 2017; Zhang and Wang, 2020) have shown that XBXT combined with antiemetics might be more conducive to reducing vomiting (RR 1.35, 95% CI: 1.25–1.46). This review found that XBXT reduced the frequency and duration of nausea. Five studies (Li et al., 1999; Zhang et al., 2005; Guo, 2008; Liu and Wang, 2008; Tao et al., 2021) revealed that XBXT combined with antiemetics could improve the overall nausea relief efficiency (RR 1.23, 95% CI: 1.09–1.38). Three studies (Li et al., 1999; Liu and Wang, 2008; Jiang et al., 2013) showed that XBXT and DARAs evidently enhanced eating efficiency compared with DARAs (RR 1.30, 95% CI: 1.07–1.57). Four studies (Guo et al., 2005; Liu and Wang, 2008; Leng and Li, 2020; Cui et al., 2021) investigated the frequency of adverse reactions concerned with antiemetics. The adverse reaction rate in XBXT was lower than that in antiemetic drugs [7.6% (14/185) vs. 14.6% (27/185)]. The meta-analysis demonstrated that the occurrence of headache, constipation, and tiredness declined after the intervention of XBXT. It has been suggested that the combination of XBXT in clinical applications can reduce the incidence of adverse reactions. Moreover, XBXT and antiemetics markedly upgraded the quality of life compared with individual antiemetic drugs (RR = 1.37, 95% CI: 1.14–1.65, *p* = 0.0009). Subgroup analysis manifested that XBXT and antiemetics elevated the vomiting relief efficiency, regardless of acute or delayed vomiting. In addition, whether the control group was treated with 5-HT3RAs or DARAs, XBXT combined with antiemetics could improve the vomiting relief efficiency. Sensitivity analysis of vomiting relief efficiency, both vomiting patterns, nausea relief efficiency, adverse event rates, and quality of life displayed similar pooled effect size estimates and robust results. The sensitivity analysis of eating rates manifested that there was a relatively high sensitivity level, as described in the study by Tao et al. (2021). After eliminating the investigation by Tao et al. (2021), the heterogeneity was evidently decreased, and the outcomes were statistically significant. The GRADE tool was applied for the estimate, demonstrating that the evidence quality was moderate in vomiting relief efficiency, delayed vomiting, eating efficiency, adverse events, and quality of life, whereas the quality of evidence for acute vomiting and nausea relief rate was high. Thus, it can be considered that the therapeutic efficacy and safety of XBXT associated with antiemetic drugs are better than those of conventional antiemetics alone.

In addition, all experiments were published in Chinese, and most of them were conducted in China. Therefore, it is not possible to draw clear conclusions for other countries. The cancer type and malignancy degree are different, which may affect the occurrence of CINV. The abovementioned factors may have generated deviations in the consequence of this meta-analysis.

## 5 Conclusion

The meta-analysis conducted in this systemic review revealed that XBXT combined with conventional antiemetic drugs had better efficacy and safety than antiemetics alone. The quality assessment indicated that the overall criteria were scientific. In addition, the potential for publication bias was relatively low. This has manifested the characteristics and superiorities of XBXT combined with antiemetics in treating CINV, which deserves recommendation. However, there are some limitations in the conclusions of this review, which demand to be settled in future investigations. Further well-designed clinical trials with higher methodological quality, larger RCT sample sizes, and inclusion of more countries may be beneficial to demonstrate the effectiveness and safety of XBXT in treating CINV.

## Data Availability

The datasets presented in this study can be found in online repositories. The names of the repository/repositories and accession number(s) can be found in the article/Supplementary Material.

## Abbreviations

CINV, chemotherapy-induced nausea and vomiting; XBXT, Xiao-Ban-Xia-Tang; RCTs, randomized controlled trials; TCM, traditional Chinese medicine; CTZ, chemoreceptor trigger zone; DA, dopamine; 5-HT, 5-hydroxytryptamine; SP, substance P; 5-HT3RAs, 5-hydroxytryptamine-3 receptor antagonists; DARAs, dopamine receptor antagonists; PRISMA, the Preferred Reporting Items for Systematic Reviews and Meta-Analyses; RR, risk ratio; MD, mean difference; SMD, standard mean difference; CIs, confidence intervals.
