# Supplementary material for: The efficacy and safety of Xiao-Ban-Xia-Tang in the treatment of chemotherapy-induced nausea and vomiting: A systematic review and meta-analysis
Source: Front Pharmacol. 2024 Jun 12;15:1393597. doi: 10.3389/fphar.2024.1393597 (PMC11199399; doi:10.3389/fphar.2024.1393597)
Supplement: Supplementary file 2 [file DataSheet1.doc]

**Supplementary Material S2. Database and Search Strategies**

**Table 1.** Search strategy in PubMed database.

| **Number** | **Search terms** |
| --- | --- |
| #1 | (("Neoplasms"[Mesh]) OR (((((((((((((((((Tumor[Title/Abstract]) OR (Neoplasm[Title/Abstract])) OR (Tumors[Title/Abstract])) OR (Neoplasia[Title/Abstract])) OR (Neoplasias[Title/Abstract])) OR (Cancer[Title/Abstract])) OR (Cancers[Title/Abstract])) OR (Malignant Neoplasm[Title/Abstract])) OR (Malignancy[Title/Abstract])) OR (Malignancies[Title/Abstract])) OR (Malignant Neoplasms[Title/Abstract])) OR (Neoplasm, Malignant[Title/Abstract])) OR (Neoplasms, Malignant[Title/Abstract])) OR (Benign Neoplasms[Title/Abstract])) OR (Benign Neoplasm[Title/Abstract])) OR (Neoplasms, Benign[Title/Abstract])) OR (Neoplasm, Benign[Title/Abstract]))) OR (("Drug Therapy"[Mesh]) OR (((((((Therapy, Drug[Title/Abstract]) OR (Drug Therapies[Title/Abstract])) OR (Therapies, Drug[Title/Abstract])) OR (Chemotherapy[Title/Abstract])) OR (Chemotherapies[Title/Abstract])) OR (Pharmacotherapy[Title/Abstract])) OR (Pharmacotherapies[Title/Abstract]))) |
| #2 | (("Vomiting"[Mesh]) OR (Emesis[Title/Abstract])) OR ("Nausea"[Mesh]) |
| #3 | ((((Xiaobanxia Tang[Title/Abstract]) OR (Xiaobanxia Decoction[Title/Abstract])) OR (Xiaobanxia[Title/Abstract])) OR (Xiao Banxia[Title/Abstract])) OR (Xiao Ban Xia[Title/Abstract]) |
| #4 | #1 AND #2 AND #3 |

**Table 2.** Search strategy in Embase database.

| **Number** | **Search terms** |
| --- | --- |
| #1 | 'neoplasm'/exp |
| #2 | 'acral tumor':ab,ti OR 'acral tumour':ab,ti OR neoplasia:ab,ti OR neoplasms:ab,ti OR 'neoplastic disease':ab,ti OR 'neoplastic entity':ab,ti OR 'neoplastic mass':ab,ti OR tumor:ab,ti OR 'tumoral entity':ab,ti OR 'tumoral mass':ab,ti OR 'tumorous entity':ab,ti OR 'tumorous mass':ab,ti OR tumors:ab,ti OR tumour:ab,ti OR 'tumoural entity':ab,ti OR 'tumoural mass':ab,ti OR 'tumourous entity':ab,ti OR 'tumourous mass':ab,ti OR tumours:ab,ti OR neoplasm:ab,ti |
| #3 | #1 OR #2 |
| #4 | 'chemotherapy'/exp |
| #5 | chemotherapeutics:ab,ti OR chemotherapy:ab,ti |
| #6 | #4 OR #5 |
| #7 | #3 OR #6 |
| #8 | 'vomiting'/exp |
| #9 | 'cyclic vomiting':ab,ti OR 'cyclical vomiting':ab,ti OR emesia:ab,ti OR emesis:ab,ti OR 'induced vomiting':ab,ti OR vomition:ab,ti OR vomitus:ab,ti OR vomiting:ab,ti |
| #10 | #8 OR #9 |
| #11 | 'nausea'/exp |
| #12 | 'creatic nausea':ab,ti OR nauseation:ab,ti OR nausea:ab,ti |
| #13 | #11 OR #12 |
| #14 | #10 OR #13 |
| #15 | 'xiaobanxia tang':ab,ti OR 'xiaobanxia decoction':ab,ti OR xiaobanxia:ab,ti OR 'xiao banxia':ab,ti OR 'xiao ban xia':ab,ti |
| #16 | #7 AND #14 AND #15 |

**Table 3.** Search strategy in Cochrane database.

| **Number** | **Search terms** |
| --- | --- |
| #1 | MeSH descriptor: [Neoplasms] explode all trees |
| #2 | (Benign Neoplasms):ti,ab,kw OR (Neoplasms, Benign):ti,ab,kw OR (Benign Neoplasm):ti,ab,kw OR (Neoplasm, Benign):ti,ab,kw OR (Tumors):ti,ab,kw OR (Neoplasias):ti,ab,kw OR (Neoplasia):ti,ab,kw OR (Neoplasm):ti,ab,kw OR (Tumor):ti,ab,kw OR (Malignancy):ti,ab,kw OR (Cancers):ti,ab,kw OR (Neoplasms, Malignant):ti,ab,kw OR (Neoplasm, Malignant):ti,ab,kw OR (Malignant Neoplasms):ti,ab,kw OR (Malignancies):ti,ab,kw OR (Malignant Neoplasm):ti,ab,kw OR (Cancer):ti,ab,kw |
| #3 | #1 OR #2 |
| #4 | MeSH descriptor: [Drug Therapy] explode all trees |
| #5 | (Chemotherapies):ti,ab,kw OR (Pharmacotherapies):ti,ab,kw OR (Drug Therapies):ti,ab,kw OR (Pharmacotherapy):ti,ab,kw OR (Chemotherapy):ti,ab,kw OR (Therapies, Drug):ti,ab,kw OR (Therapy, Drug):ti,ab,kw |
| #6 | #4 OR #5 |
| #7 | #3 OR #6 |
| #8 | MeSH descriptor: [Vomiting] explode all trees |
| #9 | (Emesis):ti,ab,kw |
| #10 | #8 OR #9 |
| #11 | MeSH descriptor: [Nausea] explode all trees |
| #12 | #10 OR #11 |
| #13 | (Xiaobanxia Tang):ti,ab,kw OR (Xiaobanxia Decoction):ti,ab,kw OR (Xiaobanxia):ti,ab,kw OR (Xiao Banxia):ti,ab,kw OR (Xiao Ban Xia):ti,ab,kw |
| #14 | #7 AND #12 AND #13 |

**Table 4.** Search strategy in CNKI database.

| **Number** | **Search terms** |
| --- | --- |
| #1 | 化疗[主题] or化学疗法[主题] or化学治疗[主题] or化学药物治疗[主题] |
| #2 | 干呕[主题] or恶心[主题] or呕吐[主题] |
| #3 | 小半夏汤[主题] or小半夏方[主题] or小半夏[主题] |
| #4 | #1 and #2 and #3 |

**Table 5.** Search strategy in VIP database.

| **Number** | **Search terms** |
| --- | --- |
| #1 | 化疗[任意字段] or化学疗法[任意字段] or化学治疗[任意字段] or化学药物治疗[任意字段] |
| #2 | 干呕[任意字段] or恶心[任意字段] or呕吐[任意字段] |
| #3 | 小半夏汤[任意字段] or小半夏方[任意字段] or小半夏[任意字段] |
| #4 | #1 and #2 and #3 |

**Table 6.** Search strategy in WanFang database.

| **Number** | **Search terms** |
| --- | --- |
| #1 | 化疗[全部字段] or化学疗法[全部字段] or化学治疗[全部字段] or化学药物治疗[全部字段] |
| #2 | 干呕[全部字段] or恶心[全部字段] or呕吐[全部字段] |
| #3 | 小半夏汤[全部字段] or小半夏方[全部字段] or小半夏[全部字段] |
| #4 | #1 and #2 and #3 |

**Table 7.** Search strategy in ClinicalTrials.gov database.

| **Filters** | **Search terms** |
| --- | --- |
| Condition/disease | neoplasm or cancer or tumor or drug therapy or chemotherapy or pharmacotherapy |
| Other terms | vomiting or emesis or nausea |
| Intervention/treatment | Xiaobanxia or Xiao Banxia or Xiao Ban Xia |
| Study Status | All studies |

**Table 8.** Search strategy in Chinese Clinical Trial Registry.

| **Retrieval experiment** | **Search terms** |
| --- | --- |
| 研究疾病名称 | 化疗后恶心 |
| 干预措施 | 小半夏 |

**Supplementary Material S3. Literature excluded after reading the full text and reasons**

**1) Combine with other intervention:**

[1] Hu, W. Y. (2011). Clinical observation on the treatment of vomiting after chemotherapy for gastrointestinal tumors with Zuozhu Daxi combined with Huangqi Injection and Xiaobanxia Pill. Jilin Med. 32(8):1500-1501. doi: 10.3969/j.issn.1004- 0412.2011.08.028.

[2] Li, Q., and Li, Y. Y. (2019). The effect of Zusanli acupoint injection combined with Xiaobanxia decoction on chemotherapy induced vomiting and quality of life after upper gastrointestinal reconstruction. Mod. J. Integr. Tradit. Chin. West. Med. 28(9):975-977,1010. doi:10.3969/j.issn.1008-8849.2019.09.017.

**2) Medical record:**

[1] Chen, S. K. (1994). Experience of Xiaobanxia Tang in treating vomiting caused by chemotherapy. Sichuan Tradit. Chin. Med. (2), 18-19.

[2] Sun, L. Y., and Li, D. P. (2000). Xiaobanxia Tang in the treatment of vomiting caused by cancer chemotherapy: A Case Study. Heilongjiang J. Tradit. Chin. Med. (4), 45.

[3] Zhang, H., Tian, J. F., Zheng, J., Ren, Q. Y. and Yang, J. G. (2017). Analysis and experience of treating chemotherapy related nausea and vomiting with Xiaobanxia and Fuling decoction. J. Emerg. Tradit. Chin. Med. 26(6), 1124-1125+1128. doi: 10. 3969/j.issn.1004-745X.2017.06. 063

**3) Not XBXT therapy:**

[1] Cui, Y. D. (2018). Observation on the therapeutic effect of Xiaobanxia Tang with modifications in preventing and treating nausea and vomiting after tumor chemotherapy in 40 cases. Hunan J. Tradit. Chin. Med. 34 (12), 49-50. doi:10.16808/ j.cnki.issn1003-7705.2018.12.020

[2] Ding, J., Zhang, B., He, G. N. (2020). Clinical observation on the treatment of cancer chemotherapy vomiting with modified Xiaobanxia Fuling Decoction. J. Emerg. Tradit. Chin. Med. 29(12),2183-2186. doi:10.3969/j.issn.1004-745X.2020.12.033

[3] Qian, Y. L. (2000). Compound Banxia Oral Liquid against chemotherapy induced vomiting. Hubei J. Tradit. Chin. Med. 22(9),38. doi:10.3969/j.issn.1000-0704.2000.09. 035

[4] Qian, Y. L., Ao, M. Z., and Xiong, J. P. (2000). Clinical and experimental observation of Compound Banxia Oral Liquid in treating vomiting caused by malignant tumor chemotherapy. Chin. J. Integr. Tradit. Chin. West. Med. Spleen Stomach. 8(5), 285-286. doi:10.3969/j.issn.1671-038X.2000.05.011

[5] Tan, Z. Y., Liu, A., and Tan, Z. Z. (2004). Study on the efficacy and safety of Sheng Banxia Tang in treating chemotherapy vomiting. Shandong J. Tradit. Chin. Med. 23(7), 410-412. doi:10.3969/j.issn.0257-358X.2004.07.014

[6] Xu, S., Li, H., Song, Y. H., Li, B. and Cai, H. B. (2009). Clinical comparison of traditional Chinese medicine compound combined with tropisetron in the prevention and treatment of gastrointestinal reactions after chemotherapy. Cancer Prev. Res.36(9), 787-790.doi:10.3971/j.issn.1000-8578.2009.09.021

[7] Zhou, Y. Y., Li, J., and Shi, H. Y. (2020). Clinical efficacy of modified Xiaobanxia decoction in preventing and treating nausea and vomiting in cancer patients after chemotherapy. Electronic J. Clin. Med. Literature. 7(8), 74.doi:10.16281/j.cnki.jocml. 2020.08.066

[8] Li, Z., Ren, H. B., Li, Y. H., Yang, L. R., Deng, B., and Yang, L. (2021). The application value of modified Xiaobanxia plus Fuling decoction assisted ondansetron in nausea and vomiting caused by chemotherapy after breast cancer surgery. J. Clin. Res. 38(11), 1727-1729. doi:10.3969/j.issn.1671-7171.2021.11.036

[9] Wu, Z. B., and Ma, Y. P. (2018). The effect of Xiaobanxia plus Fuling Decoction on preventing chemotherapy-induced vomiting. Dietetic Health-care. 5(10),63-64. doi: 10.3969/j.issn.2095-8439.2018.10.073

[10] Shi, G. M. (2010). 30 cases of prevention of chemotherapy induced gastrointestinal reactions with Xiaobanxia and Fuling Decoction. Henan Tradit. Chin. Med. 30 (7), 636. doi:10.16367/j.issn.1003-5028.2010.07.011

[11] Zhang, X. L. (2009). 30 Cases of prevention of gastrointestinal reactions caused by postoperative chemotherapy in colorectal cancer by modified Xiaobanxia Tang combined with azazetron. Chin. J. Ethnomedicine Ethnopharmacy. 18(5),85. doi:10. 3969/j.issn.1007-8517.2009.05.056

[12] Liu, S. M. (2018). Clinical study on the treatment of nausea and vomiting caused by NSCLC chemotherapy with modified Xiaobanxia Tang. [Dissertation]. Xinjiang: Xinjiang Medical University.

**4) Not RCT:**

[1] Bai, Y. J., Zhang, X. H., Wang, J. H., Yang, G. Y., Xiao, D. Y., Wang, S. H., et al. (2005). Observation on the therapeutic effect of Banxia Fuling Capsule on chemotherapy induced antiemesis in patients with malignant tumors. Acta Academiae Medicinae Zunyi. 28(4), 348-350.

[2] Tang, Y. (2018). Clinical study on the prevention and treatment of chemotherapy related delayed nausea and vomiting with Xiaobanxia Fuling Tang. [Dissertation]. Guizhou: Zunyi Medical College.

**5) Non-eligible evaluation of outcomes:**

[1] Hu, Z. R., and Zhao, Y. X. (2003). Observation on the control of delayed vomiting caused by chemotherapy by the combination of Chinese and Western medicines. J. Pract. Tradit. Chin. Med. 19(9),482. doi:10.3969/j.issn.1004-2814.2003.09.042

**6) Erroneous data:**

[1] Liu, A. H., and Yang, Y. L. (2004). Observation on the therapeutic effect of granisetron combined with and Xiaobanxia Tang in preventing vomiting caused by chemotherapy drugs. Shandong Med. J. 44(23),72-73. doi:10.3969/j.issn.1002-266X. 2004.23.085

[2] Zhang, X. L. (2008). The effect of Xiaobanxia plus Fuling Decoction on gastrointestinal hormones in chemotherapy patients. [Dissertation]. Guizhou: Zunyi Medical College.

**Supplementary Material S4. Egger’s test**

4.1 Egger’s test of vomiting relief efficiency

**Supplementary Material S5. Trim and fill analysis**

5.1 The results of trim and fill analysis of vomiting relief efficiency

5.2 The funnel plot of trim and fill analysis of vomiting relief efficiency


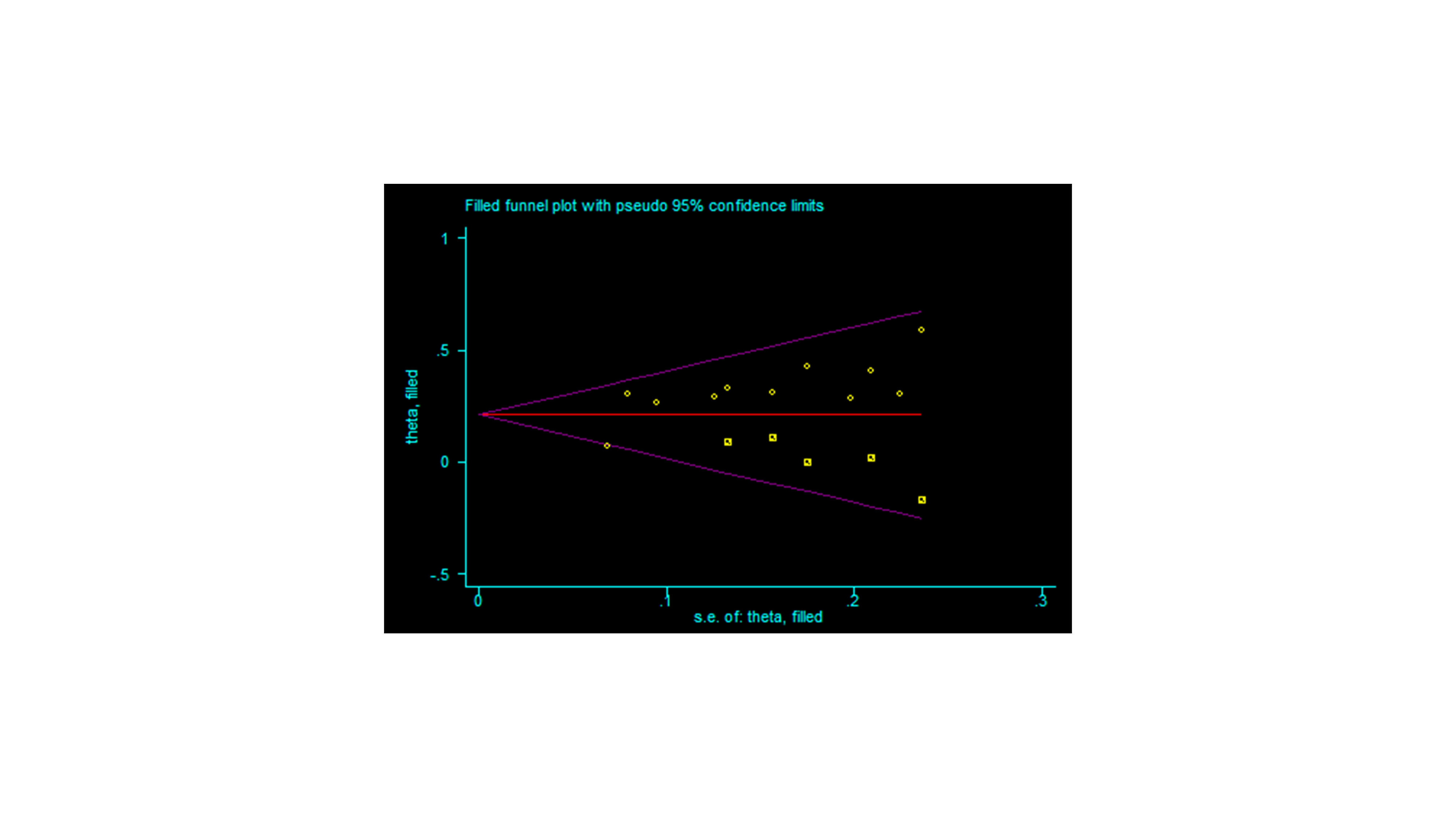


**Supplementary Material S6. Sensitivity analysis**

6.1 The results of sensitivity analysis of vomiting relief efficiency.

6.2 The results of sensitivity analysis of vomiting relief efficiency for acute vomiting.

6.3 The results of sensitivity analysis of vomiting relief efficiency for delayed vomiting.

6.4 The results of sensitivity analysis of nausea relief efficiency.

6.5 The results of sensitivity analysis of eating efficiency.

6.6 The forest plot of eating efficiency after excluding Tao 2021.


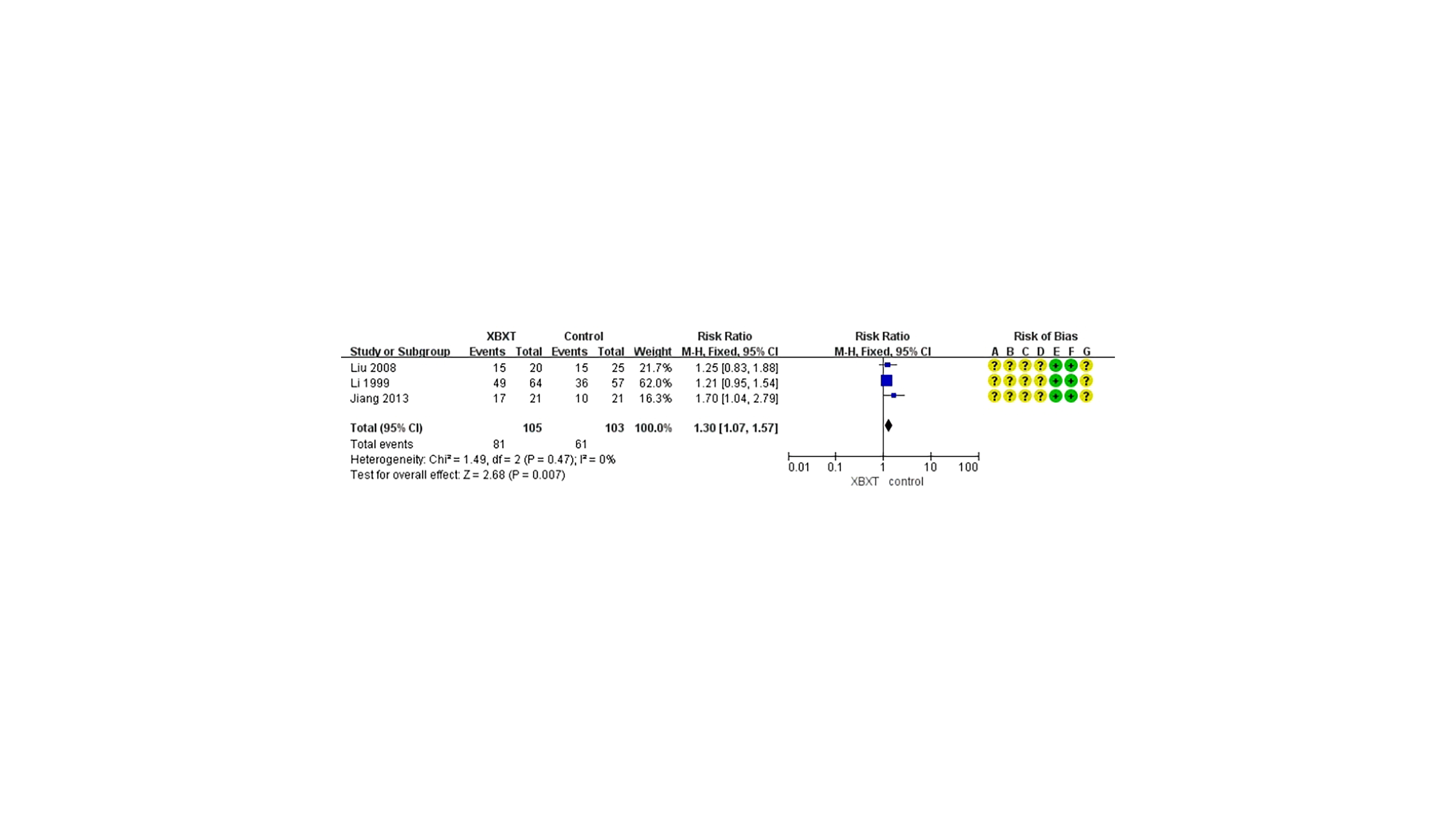


6.7 The results of sensitivity analysis of adverse events.

6.8 The results of sensitivity analysis of quality of life.
